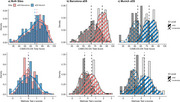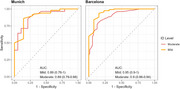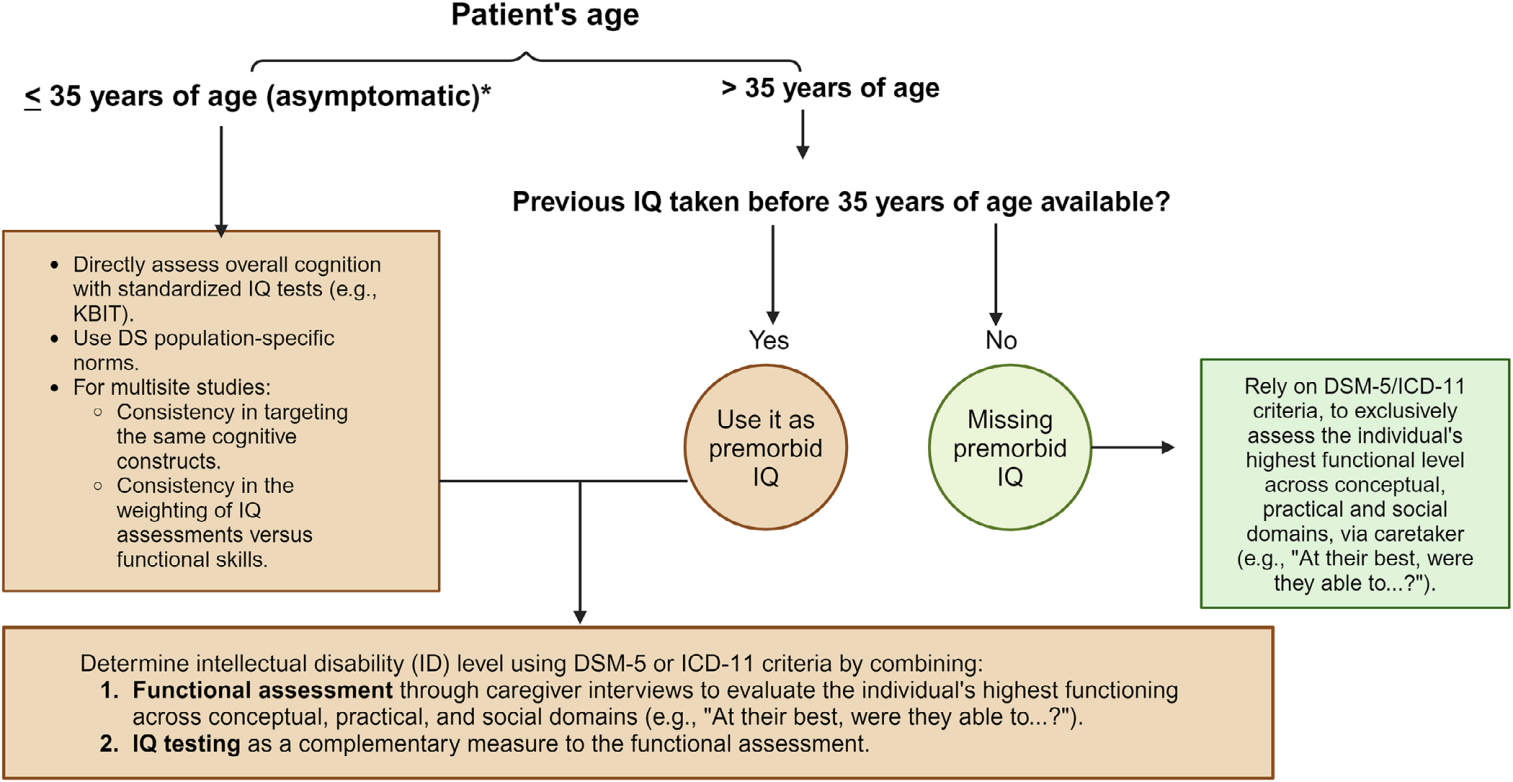# Discrepancies in Assessing Intellectual Disability Levels in Adults with Down syndrome: Implications for Dementia Diagnosis

**DOI:** 10.1002/alz70857_099293

**Published:** 2025-12-24

**Authors:** Laura Del Hoyo, Katja Sandkühler, Laura Videla, Bessy Benejam, Maria Carmona‐Iragui, Elisabeth Wlasich, Isabel Barroeta, Lídia Vaqué‐Alcázar, Íñigo Rodríguez‐Baz, Alexandre Bejanin, Susana Fernandez, Javier Arranz, José Enrique Arriola‐Infante, Lucía Maure‐Blesa, Aida Sanjuan Hernandez, Georg Nübling, Olivia Wagemann, Anna Stockbauer, Jason J. Hassenstab, Johannes Levin, Juan Fortea

**Affiliations:** ^1^ Sant Pau Memory Unit, Department of Neurology, Hospital de la Santa Creu i Sant Pau, Biomedical Research Institute Sant Pau, Barcelona, Spain; ^2^ CIBERNED, Network Center for Biomedical Research in Neurodegenerative Diseases, National Institute of Health Carlos III, Madrid, Spain; ^3^ Department of Neurology, Ludwig‐Maximilians‐Universität (LMU) München, Munich, Germany; ^4^ IIB‐Sant Pau, Hospital de la Santa Creu i Sant Pau, Universitat Autonoma de Barcelona, Barcelona, Spain; ^5^ Barcelona Down Medical Center, Fundació Catalana Síndrome de Down, Barcelona, Spain; ^6^ Sant Pau Memory Unit, Hospital de la Santa Creu i Sant Pau, Institut de Recerca Sant Pau ‐ Universitat Autònoma de Barcelona, Barcelona, Spain; ^7^ Center for Biomedical Investigation Network for Neurodegenerative Diseases (CIBERNED), Madrid, Spain; ^8^ Ludwig‐Maximilians‐Universität München, Munich, Germany; ^9^ Sant Pau Memory Unit, Department of Neurology, Institut d’Investigacions Biomèdiques Sant Pau‐Hospital de Sant Pau, Universitat Autònoma de Barcelona, Barcelona, Spain; ^10^ Department of Medicine, Faculty of Medicine and Health Sciences, Institute of Neurosciences, University of Barcelona, Barcelona, Spain. Institut d’Investigacions Biomèdiques August Pi i Sunyer (IDIBAPS), Barcelona, Spain; ^11^ Sant Pau Memory Unit, Hospital de la Santa Creu i Sant Pau, Biomedical Research Institute Sant Pau, Universitat Autònoma de Barcelona, Barcelona, Spain; ^12^ German Center for Neurodegenerative Diseases (DZNE), Munich, Bavaria, Germany; ^13^ University Hospital, LMU Munich, Munich, Bavaria, Germany; ^14^ Department of Neurology, University Hospital of Munich, LMU Munich, Munich, Germany, Munich, Germany; ^15^ Washington University in St. Louis, St. Louis, MO, USA; ^16^ Department of Neurology, LMU University Hospital, LMU Munich, Munich, Munich, Germany; ^17^ Munich Cluster for Systems Neurology (SyNergy), Munich, Munich, Germany; ^18^ Centre Médic Down de la Fundació Catalana Síndrome de Down, Barcelona, Spain

## Abstract

**Background:**

Cut‐offs derived from baseline cognitive assessments, stratified by intellectual disability (ID) level, have been proposed to diagnose symptomatic Alzheimer's disease (AD) in Down syndrome (DS). However, discrepancies in ID classification risk misclassification when applying cut‐offs across sites.

**Method:**

This dual‐center cohort study included 673 adults with mild to moderate ID at different AD stages. We assessed ID classification discrepancies across sites and its impact on CAMCOG‐DS cut‐offs for AD dementia diagnosis derived from ROC analysis.

**Result:**

Inter‐rater agreement for ID level classification was 95% within sites but 60% between sites. While CAMCOG‐DS score distributions in the whole cohort were similar across sites, ID classification discrepancies caused higher cut‐offs in Barcelona for mild and moderate ID compared to Munich. Applying site‐specific cut‐offs to another cohort reduced sensitivity and specificity.

**Conclusion:**

Standardizing ID classification is critical for generalizable cut‐offs to accurately diagnose AD dementia based on neuropsychological assessments in DS.